# Dietary Patterns and Pancreatic Cancer Risk: A Meta-Analysis

**DOI:** 10.3390/nu9010038

**Published:** 2017-01-05

**Authors:** Pei-Ying Lu, Long Shu, Shan-Shan Shen, Xu-Jiao Chen, Xiao-Yan Zhang

**Affiliations:** 1 Department of Geriatrics, Zhejiang Hospital, Xihu District, Hangzhou 310013, China; lupy007@sina.com (P.-Y.L.); shenshan305@163.com (S.-S.S.); 2 Department of Nutrition, Zhejiang Hospital, Xihu District, Hangzhou 310013, China; shulong19880920@126.com (L.S.); zxy19740804@sina.com (X.-Y.Z.)

**Keywords:** dietary patterns, alcohol consumption, pancreatic cancer, meta-analysis

## Abstract

A number of studies have examined the associations between dietary patterns and pancreatic cancer risk, but the findings have been inconclusive. Herein, we conducted this meta-analysis to assess the associations between dietary patterns and the risk of pancreatic cancer. MEDLINE (provided by the National Library of Medicine) and EBSCO (Elton B. Stephens Company) databases were searched for relevant articles published up to May 2016 that identified common dietary patterns. Thirty-two studies met the inclusion criteria and were finally included in this meta-analysis. A reduced risk of pancreatic cancer was shown for the highest compared with the lowest categories of healthy patterns (odds ratio, OR = 0.86; 95% confidence interval, CI: 0.77–0.95; *p* = 0.004) and light–moderate drinking patterns (OR = 0.90; 95% CI: 0.83–0.98; *p* = 0.02). There was evidence of an increased risk for pancreatic cancer in the highest compared with the lowest categories of western-type pattern (OR = 1.24; 95% CI: 1.06–1.45; *p* = 0.008) and heavy drinking pattern (OR = 1.29; 95% CI: 1.10–1.48; *p* = 0.002). The results of this meta-analysis demonstrate that healthy and light–moderate drinking patterns may decrease the risk of pancreatic cancer, whereas western-type and heavy drinking patterns may increase the risk of pancreatic cancer. Additional prospective studies are needed to confirm these findings.

## 1. Introduction

Pancreatic cancer is the fourth leading cause of cancer-related death in both men and women worldwide, with approximately 338,000 new cases occurring each year [1]. In Europe, pancreatic cancer is the fifth most common cause of cancer death in men and the fourth in women [2]. Although the incidence of pancreatic cancer in China is lower than that in the West, it has increased markedly in recent years, becoming a substantial burden in China [3]. It is well-known that pancreatic cancer is a multifactorial disease that results from complex interactions of some etiologic factors, including genetic factors, age, alcohol, cigarette smoking, history of diabetes mellitus and obesity, and dietary factors [4].

Over the past few decades, several epidemiological studies have specifically focused on dietary modification as an important influential factor in the development of pancreatic cancer and examined the link between food groups and individual nutrients and pancreatic cancer risk [5,6]. However, the high inter-correlation between foods and nutrients often makes it difficult to identify the effects of single dietary components [7]. Consequently, dietary pattern analysis has emerged as an approach in assessing the association between whole-diet and diseases, taking into account the combined effects of foods and potentially facilitating nutritional recommendations [8].

To date, in medical research there has been considerable attention paid to the relation between dietary patterns and pancreatic cancer risk [9,10,11]. However, the association of dietary patterns with the risk of pancreatic cancer has been inconclusive. Several studies have also reported the decreased risk of pancreatic cancer associated with nutrients commonly found in fruits and vegetables [12,13]. A study by Larsson et al. [14] has shown no significant association between intake of fruits, vegetables and risk of pancreatic cancer. A recent review by an international panel of experts also concluded that the evidence for an association of vegetable consumption in relation to pancreatic cancer risk is limited and inconsistent [15]. In a large-scale population-based cohort study in Japan, the results showed a decreased risk for pancreatic cancer when comparing the highest versus lowest intakes of coffee [16]. Besides, a population based case-control study conducted in Shanghai, China, showed a statistically significant inverse association with increased tea consumption and pancreatic cancer risk [17]. A previous meta-analysis [18] also reported an overall significant inverse association of low to moderate alcohol consumption (<3 drinks/day) and pancreatic cancer risk, compared with non-drinking. Similarly, a study by Heinen et al. [19] also reported an increased risk of pancreatic cancer for persons with a heavy alcohol intake. In the European Prospective Investigation into Cancer and Nutrition (EPIC), research has reported that the consumption of red and processed meat are not associated with an increased risk of pancreatic cancer, while the consumption of poultry is associated with an increased pancreatic cancer risk [20]. However, to our knowledge, in the update report about pancreatic cancer by World Cancer Research Fund (WCRF) and the American Institute for Cancer Research (AICR), no firm judgment has been made on the relation between red and processed meat and the risk of pancreatic cancer [4]. We therefore conducted a systematic meta-analysis of studies published up to May 2016, to assess the potential associations of dietary patterns with pancreatic cancer risk.

## 2. Methods

### 2.1. Literature Search Strategy

An electronic literature search was performed in MEDLINE (provided by the National Library of Medicine) and EBSCO (Elton B. Stephens Company) to identify relevant studies written in the English and Chinese languages published up to May 2016, with the following keywords or phrases: “dietary pattern” OR “dietary patterns” OR “eating pattern” OR “eating patterns” OR “food pattern” OR “food patterns” OR “diet” OR “alcohol drinking” OR “alcohol consumption” AND “pancreatic cancer” OR “pancreatic neoplasm” OR “pancreatic carcinoma” OR “cancer of pancreatic”. Moreover, we searched the references lists of retrieved articles to identify further studies.

### 2.2. Studies Included Criteria

Three independent reviewers read the abstracts of papers retrieved in the initial search to identify studies that examined the relationship between dietary patterns and pancreatic cancer risk. Differences between the three reviewers were resolved by consensus and referred to the four reviewers if necessary. When all reviewers agreed, the full-text versions of articles were reviewed against inclusion and exclusion criteria for the present meta-analysis. To be eligible, the studies had to fulfill the following criteria: (1) The study was an original report investigating the relation between dietary patterns and pancreatic cancer risk; (2) Factor analysis and/or principal component analysis was used to identify food patterns; (3) Odds ratios and percentage of pancreatic cancer (or sufficient information to calculate them) had been listed; (4) If the data in original publication lacked sufficient details, the corresponding author of the study was contacted for additional information by email; (5) Pancreatic cancer diagnoses were confirmed by the clinical manifestations, endoscopic ultrasonography and pathological section.

### 2.3. Data Extraction

The following data were extracted from each publication: the first author’s last name, year of publication, country where the study was performed, study design, sample size, number of pancreatic cancer, dietary assessment method, identification of dietary patterns and the variables adjusted for in the present analysis.

### 2.4. Definition of “High Intake”

The different forms of alcohol intake were converted into grams of ethanol per day. Alcohol consumption < 12.5 g/day (1 drink/day) for men or 7.5 g/day (0.5 drinks/day) for women was defined as a low alcohol intake; alcohol consumption > 50 g/day (4 drinks/day) for men or 25 g/day (2 drinks/day) for women was defined as a high alcohol intake, and alcohol consumption > 12.5 g/day (1 drinks/day) and <50 g/day (4 drinks/day) for men or >7.5 g/day (0.5 drinks/day) and <25 g/day (2 drinks/day) for women was defined as a light-moderate alcohol intake [21].

### 2.5. Assessment of Heterogeneity

The Cochran’s Q statistic and *I*^2^ statistic were used to evaluate heterogeneity. A *p* value of Q-test > 0.10 or *I*^2^ < 50% indicated an absence of heterogeneity between studies, and a fixed-effects model (Mantel–Haenszel method) was used to calculated the pooled odds ratios (ORs). If a *p* value of Q-test ≤ 0.10 or *I*^2^ ≥ 50% indicated a high degree of heterogeneity among studies, then a random-effects model (DerSimonnian and Laird method) was used [22].

### 2.6. Quality Assessment

The reviewers independently assessed the risk of bias using the Newcastle–Ottawa Quality Assessment scale for studies included in this meta-analysis [23]. A maximum of nine points was assigned to each study. Only these studies which the majority of the questions were deemed satisfactory (i.e., with a score of 6 or higher) were considered to be of high methodological quality.

## 3. Statistical Analysis

Statistical analyses were performed by using Review Manager, version 5.0 (Nordic Cochrane Centre, Copenhagen, Denmark) and STATA, version 12 (Stata Corp, College Station, Texas City, TX, USA). The original studies reported the results of dietary patterns in terms of tertiles, quartiles, and quintiles of dietary factor scores and pancreatic cancer risk. We conducted this meta-analysis to assess the risk of pancreatic cancer in the highest versus the lowest categories of healthy, western-type, heavy drinking and light-moderate drinking patterns. Multivariable adjusted odds ratios, hazards ratios and relative risks with 95% confidence intervals (CIs) from individual studies were combined to produce an overall OR. Publication bias was assessed by inspection of the funnel plot and by formal testing for “funnel plot” asymmetry using Begg’s test and Egger’s test [24]. Sensitivity analysis was conducted to determine whether differences in age, sample size, races and study design affected the conclusions. All statistical tests were two-sided and *P* values less than 0.05 were considered significant.

## 4. Results

### 4.1. Overview of Included Studies for the Systematic Review

**Figure 1:**
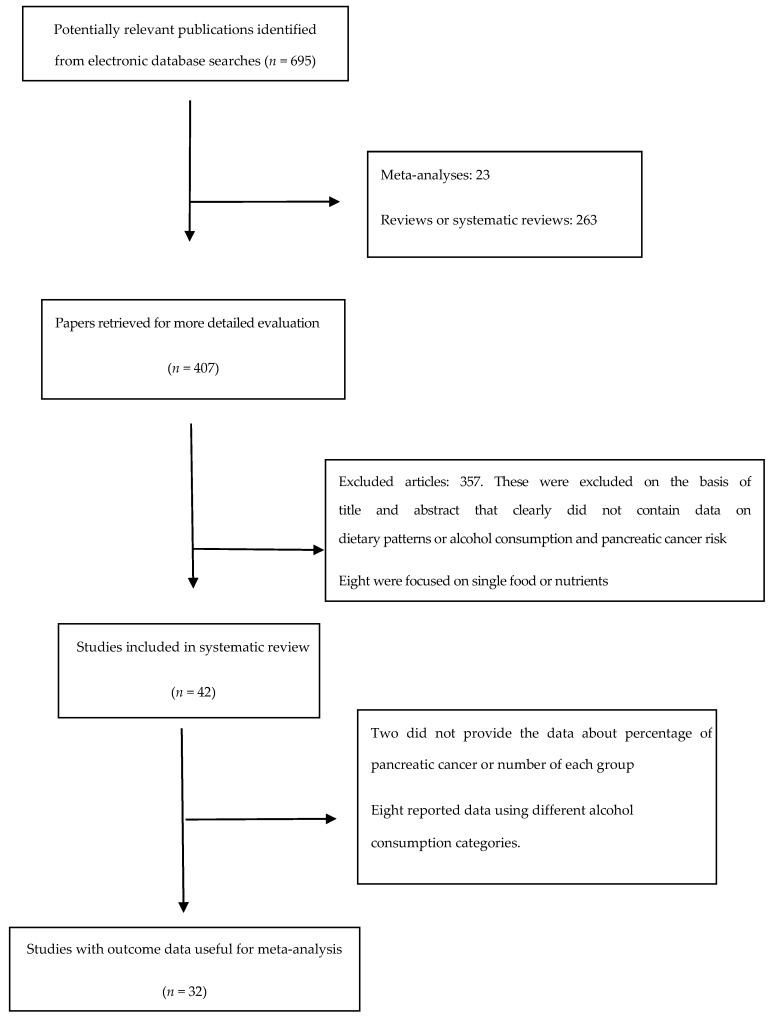
Flow chart of article screening and selection process.

An electronic literature search in the database of MEDLINE and EBSCO identified 695 studies, 663 of which were excluded based on the reasons listed in Figure 1. Thirty-two articles [9,10,11,14,19,25,26,27,28,29,30,31,32,33,34,35,36,37,38,39,40,41,42,43,44,45,46,47,48,49,50,51] met the inclusion criteria and were included in this meta-analysis, including 18 [11,14,19,25,26,28,30,32,34,35,36,37,39,40,47,49,50,51] cohort studies and 14 [9,10,27,29,31,33,38,41,42,43,44,45,46,48] case-control studies. Study characteristics are presented in Table 1.

### 4.2. Healthy Pattern

**Figure 2:**
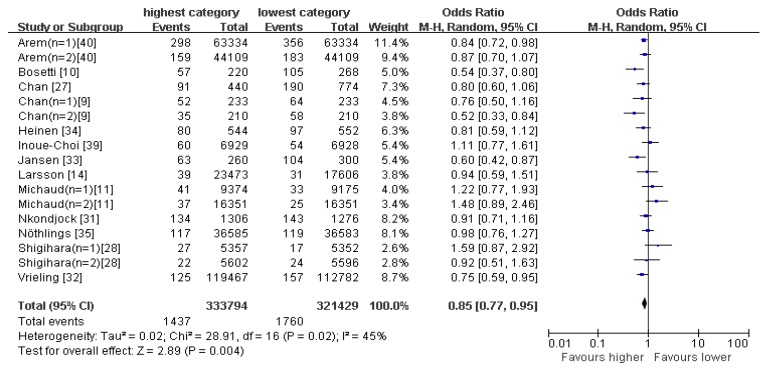
Forest plot for odds ratios (ORs) of the highest compared with the lowest category of intake of the healthy pattern and pancreatic cancer. CI: confidence interval.

The healthy pattern is characterized to have high loadings of foods such as vegetables, fruits, whole grains, olive oil, fish, soy, poultry and low-fat dairy. The relation between healthy pattern and pancreatic cancer risk is shown in Figure 2. There was evidence of a reduced risk of pancreatic cancer in the highest compared with the lowest category of healthy pattern (OR = 0.85; 95% CI: 0.77–0.95; *p* = 0.004), where all studies were combined in the random-effects model. The heterogeneity was apparent in all the studies (*p* = 0.02; *I*^2^ = 45%).

### 4.3. Western-Type Pattern

**Figure 3:**
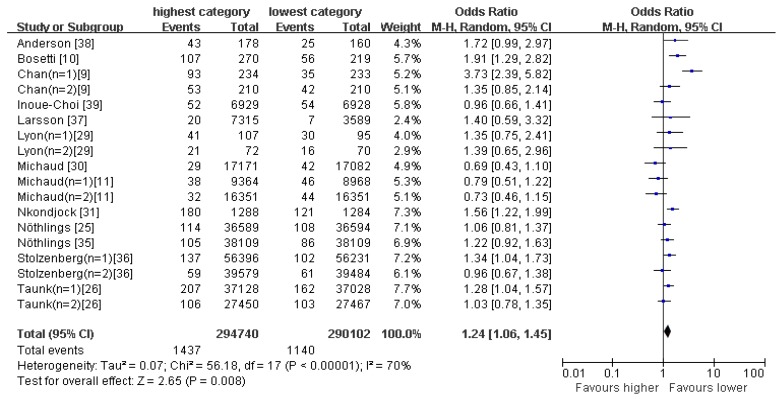
Forest plot for ORs of the highest compared with the lowest category of intake of the western-type pattern and pancreatic cancer.

The western-type pattern is characterized to have high consumption of e.g., red and/or processed meat, refined grains, sweets, high-fat dairy products, butter, potatoes and high-fat gravy, and low intakes of fruits and vegetables. Figure 3 shows the forest plot for the risk of pancreatic cancer in the highest compared with the lowest category of western-type pattern. There was significant heterogeneity (*I*^2^ = 70%, *p* < 0.00001) and hence the effect was assessed using the the random-effects model. The results demonstrated that western-type pattern was associated with an increased risk of pancreatic cancer (OR = 1.24; 95% CI: 1.06–1.45; *p* = 0.008).

### 4.4. Drinking Pattern

**Figure 4:**
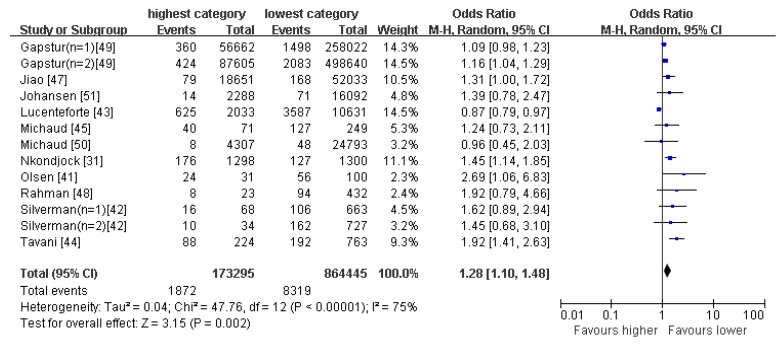
Forest plot for ORs of the highest compared with the lowest category of intake of the heavy drinking pattern and pancreatic cancer.

**Figure 5:**
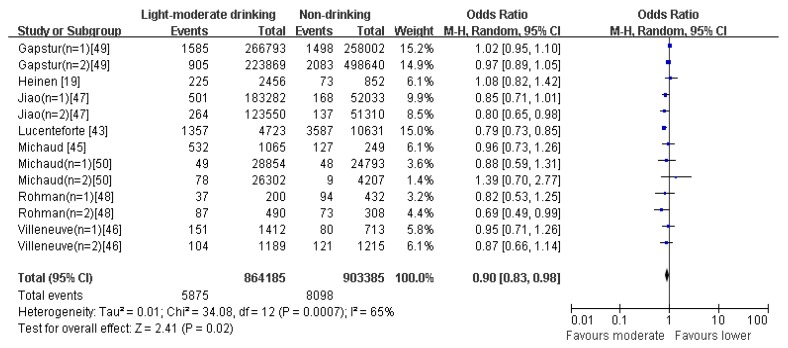
Forest plot for ORs of light–moderate drinking compared with non-drinking intake of the light–moderate drinking pattern and pancreatic cancer.

The drinking pattern is characterized to have high loadings of beers, wines, and white spirits. Eleven articles reporting thirty original studies were identified as, (or to include the) heavy drinking pattern in this meta-analysis (Figure 4). There was evidence of an increased risk of pancreatic cancer in the highest compared with the lowest category of heavy drinking pattern (OR = 1.28; 95% CI: 1.10–1.48; *p* = 0.002). Data from these studies were assessed using a random-effects model, and there was obvious evidence of heterogeneity (*p* < 0.00001; *I*^2^ = 75%). Pooled results from eight articles identified a light-moderate drinking pattern. Figure 5 showed an obvious evidence of a decreased risk of pancreatic cancer in the light–moderate drinking compared with non-drinking (OR = 0.90; 95% CI: 0.83–0.98; *p* = 0.02). Data from these studies were assessed using random-effects model, and there was obvious evidence of heterogeneity (*p* = 0.0007; *I*^2^ = 65%).

### 4.5. Publication Bias

Funnel plots revealed little evidence of asymmetry, and thus little evidence of publication bias (highest compared with lowest categories: healthy pattern Begg’s test *p* = 0.275; Western-type pattern Begg’s test *p* = 0.386; heavy drinking pattern Begg’s test *p* = 0.218; and light-moderate drinking pattern Begg’s test *p* = 0.351).

### 4.6. Quality Assessment

The quality of each study in terms of population and sampling methods, description of exposure and outcomes, and statistical adjustment of data, is summarized in Table A1. Of the 32 studies, 26 received a score of 6 or higher on the Newcastle-Ottawa Quality assessment scale and were considered to be of high methodological quality [9,10,11,14,19,25,26,27,28,30,31,32,34,35,36,37,39,40,42,45,46,47,48,49,50,51].

### 4.7. Sensitivity Analysis

The sensitivity analysis revealed that differences in age, sample size, race and study design had an impact on the link between dietary patterns and pancreatic cancer risk. When the highest category was compared with the lowest category of healthy pattern, the healthy pattern/pancreatic cancer association was obvious when sample size was less than 5000, study design was case-control and subjects were white and more than 50 years old. When the results were analyzed by removing cohort studies and those with age less than 50 years old, the positive relationship between western-type pattern and pancreatic cancer was more obvious. In addition, the positive association was obvious for those in the highest compared with the lowest category of heavy drinking pattern in studies with a small sample size where the subjects were white and more than 50 years old. Furthermore, the inverse association was obvious for those in the highest compared with the lowest category of light–moderate drinking pattern in studies with a large sample size, case-control design and where the subjects were more than 50 years old. After careful analysis, we found that the factor of pack/years of smoking is difficult to include in this sensitivity analysis. The reason is that the smoking variable is different in the included studies. It is difficult to distinguish the its effect on the relationship between dietary patterns and pancreatic cancer risk. However, we will pay attention to this problem in the following prospective study. In a word, as these variables have a strong effect on the association between different dietary patterns and pancreatic cancer risk, their differences may partially explain the heterogeneity between studies (Table 2).

## 5. Discussion

To our knowledge, this is the first meta-analysis reporting the associations between different dietary patterns and pancreatic cancer risk. The results indicate that healthy and light-moderate drinking patterns may decrease the risk of pancreatic cancer; whereas western-type and heavy drinking patterns may increase the risk of pancreatic cancer. Data from 32 studies involving 4,803,601 participants were included in our analyses. In the World Cancer Research Fund or American Institute For Cancer Research (WCRF/AICR) report published in 2012, there is limited evidence suggesting that red meat and alcohol intake are risk factors for pancreatic cancer. Our findings add to the existing literature and provide a strong support to the concept that diet is significantly associated with pancreatic cancer risk.

In this meta-analysis, we observed an inverse association between healthy pattern and pancreatic cancer risk. Some previous studies reported the favorable effect of fruit and vegetables intake on the prevention of pancreatic cancer [37,42,43]. The protective effect of vegetables and fruits against pancreatic cancer may be plausible due to their high content of antioxidant substances (e.g., vitamin C, vitamin E, carotenoids, phenols, and flavonoids) and dietary fiber. It is acknowledged that vitamin C can protect cells from oxidative DNA damage, thereby blocking carcinogenesis [52]. In addition, antioxidants such as vitamin C/E have an effect on the inflammatory process, particularly chronic inflammatory processes, which may play an important role in pancreatic carcinogenesis [53]. Furthermore, previous studies have also found that high dietary fiber consumption is associated with a decreased risk of pancreatic cancer [54]. Although the exact biologic mechanisms remain unclear, dietary fiber may act as a cancer preventive, for example by lowering the levels of circulating markers of inflammation, which are involved in pancreatic cancer initiation and progression. They also improve insulin metabolism by modulating hormonal pathways linked to pancreatic carcinogenesis [55,56]. Finally, vegetables and fruits contain large amounts of folate. A previous meta-analysis has reported that dietary folate plays a protective role in carcinogenesis of pancreatic cancer [57].

The western-type pattern was associated with an increased risk of pancreatic cancer. Our findings were consistent with results from previous studies [9,10], indicating that western and/or animal food pattern can increase the risk of pancreatic cancer. When cooking at high temperatures, red meat may contain heterocyclic amines and polycyclic aromatic hydrocarbons, which are considered carcinogenic [58]. Moreover, high red meat consumption may result in more absorption of haem iron, greater oxidative stress, and potential for DNA damage [59]. Several randomized controlled trails also found that saturated fatty acids were significantly associated with insulin resistance and diabetes, which are risk factors for pancreatic cancer [60]. Furthermore, processed meats are usually preserved with nitrite and may contain *N*-nitroso compounds and heterocyclic amines. Experimental studies found that *N*-nitroso compounds and heterocyclic amines were potent carcinogens that may induce pancreatic cancer [61].

The heavy drinking pattern was associated with an increased risk of pancreatic cancer in our analyses. A published meta-analysis of alcohol consumption and pancreatic cancer risk reported that heavy alcohol intake was associated with an increased risk of pancreatic cancer [18]. In fact, alcohol consumption has been consistently recognized as an important carcinogen. As far as we know, there are some plausible explanations for this relationship. Firstly, acetaldehyde, the main metabolite of ethanol, is a known human carcinogen [62]. Secondly, fatty acid esters, products of the interaction between ethanol and fatty acids, accumulate in the pancreas and could induce inflammatory response, fibrosis and thus contribute to pancreatic carcinogenesis [63]. Third, alcohol intake is an important determinant of chronic pancreatitis, a known risk factor for pancreatic cancer [64]. Furthermore, heavy alcohol consumption may also increase production of reactive oxygen species which may result in oxidative DNA damage and dysregulation of proliferation and apoptosis [65]. However, we also observed an inverse association of light-moderate drinking pattern and pancreatic cancer risk. A plausible explanation for a reduced risk of pancreatic cancer with moderate alcohol intake may be that moderate intake lowers the levels of fasting insulin, which is related to the decreased risk of pancreatic cancer [66,67]. A recent systematic review and meta-analysis concluded that metabolic syndrome was associated with increased risk of common cancers, including pancreatic cancer [68].

### Strengths and Limitations

This meta-analysis holds its own strengths and limitations. Firstly, this is the first meta-analysis focused on the relation between dietary patterns and pancreatic cancer risk. Besides, we also further explored the associations between heavy and light-moderate drinking patterns and pancreatic cancer risk. Secondly, pancreatic cancer diagnoses were confirmed by the clinical manifestations, endoscopic ultrasonography and pathological section, avoiding misdiagnosis. Thirdly, no signs of publication bias were evident in the funnel plot, and the statistical test for publication bias was non-significant. However, several limitations should be considered in this meta-analysis. Firstly, there was an inconsistent adjustment for potential confounders among the included studies. Consequently, the data included in our analyses might suffer from differing degrees of completeness and accuracy. Secondly, 14 of 32 studies used a case-control design, which is more susceptible to selection and recall bias, especially dietary recall bias, than a cohort design.

## 6. Conclusions

In conclusion, this meta-analysis showed that the healthy and light–moderate drinking patterns are associated with a reduced risk of pancreatic cancer, whereas the western-type and heavy drinking patterns are associated with an increased risk of pancreatic cancer. Our findings confirm the significant associations between dietary patterns and pancreatic cancer risk, and add to the existing literature supporting the concept that diet plays an important role in the prevention of pancreatic cancer. Additional prospective studies are needed to confirm the cause relationship between dietary patterns and pancreatic cancer risk.

## Figures and Tables

**Table 1:** Characteristics of 32 studies included in the meta-analysis (1989–2016).

Author Publication Year	Location	Study Design	Total Number of Subjects	Age	Diet-Assessment Method	Factors Adjusted for in Analysis	Dietary Patterns Identified
Chan et al. 2013 [9]	United States	Case-control	532 cases 1701 controls	21–85 years	FFQ	Age, race, education, diabetes, body mass index, smoking, alcohol drinking, physical activity, and total energy.	Prudent, western diet
Bosetti et al. 2013 [10]	Italy	Case-control	326 cases 625 controls	Mean: 62 years	FFQ	Age, sex, study center and adjusted for year of interview, education, body mass index, tobacco smoking, alcohol drinking, and diabetes.	Animal products, unsaturated fats, vitamins and fiber, starch rich
Michaud et al. 2005 [11]	United States	Case-control	47,493 men 77,179 women	40–75 years	FFQ	Age, pack-years of smoking (for current and past smokers in past 15 years), body mass index, physical activity, history of diabetes mellitus, caloric intake, height, and multivitamin use.	Western, prudent
Nöthlings et al. 2008 [25]	United States	Cohort	424,978	35–70 years	FFQ	Age, sex, and center as strata variables and for diabetes mellitus at baseline, BMI, energy intake, smoking status (4 categories), and the number of cigarettes as covariates.	Food; simplified food
Taunk et al. 2016 [26]	United States	Cohort	322,848	50–71 years	FFQ	Smoking, BMI, self-reported diabetes (yes, no) and energy-adjusted saturated fat (continuous).	Total meat
Chan et al. 2005 [27]	United States	Case-control	532 cases 1701 controls	21–85 years	FFQ	Age, sex, and energy intake.	Total vegetables and fruits
Shigihara et al. 2014 [28]	Japan	Cohort	32,859	40–74 years	FFQ	Age in years, body mass index in kg/m^2^, family history of cancer (yes or no), history of diabetes mellitus (yes or no), smoking status, alcohol consumption, time spent walking in hours/day (<0.5, 0.5–0.9, or ≥1), education (junior high school or less, high school, or college/ university or higher), marital status (married or unmarried), job status (employed or unemployed), consumption of meat in g/day and total caloric intake in kcal/day.	Total vegetables and fruits
Lyon et al. 1993 [29]	United States	Case-control	149 cases 2363 controls	< 65 years	FFQ	Age, cigarette smoking, and consumption of coffee and alcohol	Red meat
Michaud et al. 2003 [30]	United States	Cohort	88.802	30–55 years	FFQ	Pack-years of smoking (past 15 years; current and past smokers separately), body mass index (quintiles in 1976), history of diabetes mellitus, caloric intake (quintiles), height (quintiles), physical activity (continuous), and menopausal status.	Total meat intake
Nkondjock et al. 2005 [31]	Canada	Case-control	585 cases 4779 controls	30–74 years	FFQ	Age (in 5-year groups), smoking (0, >0–15 and >15 pack-years), BMI (5 categories), physical activity (total number of hours/month of moderate and strenuous activities), province (eight groups), educational attainment (years) and total energy intake (as a continuous variable).	Western, fruit and vegetables, drinker
Vrieling et al. 2009 [32]	European countries	Cohort	478,400	35–70 years	FFQ	Age at entry, sex and center and adjusted for energy from fat, energy from non-fat, weight, height, history of diabetes (yes, no, missing), and smoking status (never, past (quit <10 year, 10 year), current (intensity 1–14, 15–24, 25 cig/day), unknown).	Total fruit and vegetable consumption
Larsson et al. 2006 [14]	Sweden	Cohort	81,922	> 55 years	FFQ	Age (in months), sex, education (less than high school, high school graduate, or more than high school), body mass index (<23.0, 23.0–24.9, 25.0–29.9, or ≥30 kg/m^2^), physical activity (hours/week; four categories), cigarette smoking status and pack-years of smoking (never, past < 20 pack-years, past ≥ 20 pack-years, current < 20 pack-years, current 20–39 pack-years, or current 40 pack-years), history of diabetes (yes or no), multivitamin supplement use (no use, occasional use, or regular use), and intakes of total energy (continuous) and alcohol (quartiles).	Fruits and vegetable consumption
Jansen et al. 2011 [33]	United States	Case-control	384 cases 983 controls	24–94 years	FFQ	Age, sex, smoking, body mass index, energy intake, and alcohol consumption.	Fruit and vegetable intake
Heinen et al. 2012 [34]	Netherlands	Cohort	120,852	55–69 years	FFQ	Age(year), sex, smoking (current smoking: yes/no; number of cigarettes smoked per day; number of years of smoking), body mass index (kg/m^2^), family history of pancreatic cancer (yes/no), history of diabetes mellitus (yes/no), intake of energy (kcal/day), red meat (g/day), coffee (number of cups/day), and alcohol (g/day).	Fruit and vegetables
Nöthlings et al. 2005 [35]	United states	Cohort	190,545	45–75 years	FFQ	Sex and time on study and adjusted for age at cohort entry, ethnicity, history of diabetes mellitus, familial history of pancreatic cancer, smoking status, and energy intake.	Red meat intake
Stolzenberg-Solomon et al. 2007 [36]	United States	Cohort	537,302	50–71 years	FFQ	Age, energy, smoking, BMI, education, race, self- reported diabetes(yes/no), energy-adjusted saturated fat.	Total meat intake
Larsson et al. 2006 [37]	Sweden	Cohort	61,433	> 50 years	FFQ	Age (in months), education (less than high school, high school graduate, or more than high school), body mass index (<23.0, 23.0–24.9, 25.0–29.9 or 30 kg/m^2^), smoking (never smoker, past and smoked <20 pack-years, past and smoked 20 pack-years, current and smoked <20 pack-years or current and smoked 20 pack-years) and intakes of total energy (continuous), alcohol (quartiles) and energy-adjusted folate (quartiles).	Red meat
Anderson et al. 2002 [38]	United States	Case-control	193 cases 674 controls	20–64 years	FFQ	Age, sex, smoking (pack-years and pack-years squared), education, race, diabetes, white meat, red meat not grilled, and other red meat.	Meat intake
Inoue-Choi et al. 2011 [39]	United States	Cohort	34,642	55–69 years	FFQ	Age (continuous), race, education (less than high school, high school, greater than high school), alcohol intake (yes/no), smoking (current, past, never smoker), physical activity (low, moderate, high).	Mediterranean; red meat
Arem et al. 2013 [40]	United States	Cohort	537,128	50–71 years	FFQ	Daily caloric intake, sex (where appropriate), diabetes (yes/no), body mass index (15 to <18.5, 18.5 to <25, 25 to <30, 30 to ≤50 kg/m^2^, missing) and smoking status (categories describing never, ever, current, and dose).	HEI-2005
Olsen et al. 1989 [41]	United States	Case-control	212 cases 220 controls	40–84 years	FFQ	Age, education level, reported diabetes mellitus history, cigarette smoking, meat and vegetable consumption.	Total alcohol
Silverman et al. 1995 [42]	United States	Case-control	486 cases 2109 controls	30–79 years	Questionnaire	Age, area, cigarette smoking, gallbladder disease, diabetes, and income.	Total alcohol consumption
Lucenteforte et al. 2012 [43]	Europe, China, United States	Case-control	5585 cases 11,827 controls	Mean: 64 years	Questionnaire	Age, sex, race/ethnicity, education, body mass index, history of diabetes, tobacco smoking (in categories, plus a continuous term), and center for multicentric studies.	Total alcohol consumption
Heinen et al. 2009 [19]	Netherlands	Cohort	12,085	55–69 years	FFQ	Age (years), sex, smoking (smoking status (current smoking: yes/no); number of cigarettes smoked per day; number of years of smoking), energy intake (kcal/day), body mass index (weight (kg)/height (m)^2^), vegetable intake (g/day), and fruit intake (g/day).	Total ethanol intake
Tavani et al. 1997 [44]	Italy	Case-control	361 cases 997 controls	17–79 years	Questionnaire	Age, sex, education, smoking status, and history of diabetes, pancreatitis, and cholelithiasis.	Total alcohol intake
Michaud et al. 2010 [45]	Europe, China, United States	Case-control	1530 cases 1530 controls	> 60 years	Questionnaire	Age (continuous), cohort, gender, race (Caucasian, Asian, other), smoking (dose continuous, duration continuous), diabetes (yes, no, missing), and BMI (continuous).	Total alcohol
Villeneuve et al. 2000 [46]	Canada	Case-control	583 cases 4813 controls	Mean: 59 years	Questionnaire	Age, province, coffee consumption, cigarette pack-years, energy intake and dietary fat.	Total alcohol
Jiao et al. 2009 [47]	United States	Cohort	470,681	50–71 years	Questionnaire	Sex (for all); smoking variable (never smokers, quit 10 years ago and smoked <20 cigarettes/day, quit 10 years ago and smoked 20 cigarettes/day, quit 5–9 years ago and smoked <20 cigarettes/day, quit 5–9 years ago and smoked 20 cigarettes/day, quit 1–4 years ago and smoked <20 cigarettes/day, quit 1–4 years ago and smoked 20 cigarettes/day, current smokers with <20 cigarettes/day, and current smokers with 20 cigarettes/day); total energy intake (continuous), energy-adjusted saturated fat, red meat, and total folate intake (continuous scale); body mass index (<20, 20 to <25, 25 to <30, 30 kg/m^2^, missing); physical activity (low, moderate, and high level); and history of diabetes.	Alcohol use
Rahman et al. 2015 [48]	Canada	Case-control	345 cases 1285 controls	≤89 years	Questionnaire	Sex, age, body mass index (based on weight one year prior to questionnaire completion), type 2 diabetes, pancreatitis, family history of pancreas cancer, smoking status (non-smoker, current, former)	Alcohol consumption
Gapstur et al. 2011 [49]	United States	cohort	453,770 men 576,697 women	30–111 years	Questionnaire	Age, sex, race/ethnicity, education, marital status, body mass index, family history of pancreatic cancer, and personal history of gallstones, diabetes mellitus, or smoking.	Alcohol intake
Michaud et al. 2001 [50]	United States	cohort	51,529 men 121,700 women	40–75 years	FFQ	Age in 5-year categories, pack-years of smoking (past 15 years; current and past smokers separately), BMI (quintiles at baseline), history of diabetes mellitus, history of cholecysectomy, energy intake (quintiles), and period.	Alcohol intake
Johansen et al. 2009 [51]	Sweden	Cohort	33,346	Mean: 50 for men; 44 for women	Questionnaire	Age, sex, smoking status, Mm-MAST category (Mm-MAST is not adjusted for -glutamyl transferase (GT) and -GT is not adjusted for Mm-MAST) and BMI (weight gain not adjusted for BMI).	Alcohol consumption

FFQ: Food Frequency Questionnaire; HEI-2005: Healthy Eating Index 2005; Mm-MAST: Malmö modification of the brief Michigan Alcoholism Screening Test); BMI: body mass index; GT: glutamyl transferase.

**Table 2:** Dietary patterns and pancreatic cancer: sensitivity analysis.

Study Characteristic	Category	Healthy Pattern (95% CI)	Western-Type Pattern (95% CI)	Heavy Drinking Pattern (95% CI)	Light–Moderate Drinking Pattern (95% CI)
Age	>50	0.86 (0.76, 0.98)	1.23 (1.02, 1.47)	1.23 (1.11, 1.36)	0.94 (0.87, 1.00)
	<50	0.91 (0.71,1.16)	1.28 (0.91, 1.80)	1.23 (0.75, 2.02)	0.84 (0.69, 1.02)
Sample size	Large (>5000)	0.98 (0.86, 1.11)	1.14 (1.00, 1.30)	1.14 (0.98, 1.32)	0.91 (0.83, 1.00)
	Small (<5000)	0.72 (0.62, 0.85)	1.84 (1.22, 2.76)	1.73 (1.39, 2.16)	0.84 (0.69, 1.02)
Race	White	0.85 (0.75, 0.95)	1.24 (1.06, 1.45)	1.33 (1.16, 1.52)	0.94 (0.87, 1.00)
	Yellow and Other	1.20 (0.70, 2.06)	-	0.94 (0.71, 1.25)	0.84 (0.69, 1.02)
Study design	Case-control	0.70 (0.59, 0.85)	1.78 (1.36, 2.32)	1.47 (1.06, 2.04)	0.81 (0.76, 0.86)
	Cohort	0.95 (0.85, 1.07)	1.06 (0.93, 1.20)	1.14 (1.06, 1.23)	0.96 (0.89, 1.03)

## Appendix A

**Table A1:** Dietary patterns and pancreatic cancer: Assessment of Study Quality.

Studies	Selection				Comparability		Outcome			Score
	1	2	3	4	5A	5B	6	7	8	
Chan et al. 2013 [9]	*		*	*	*		*	*		******
Bosetti et al. 2013 [10]	*		*	*	*		*	*	*	*******
Michaud et al. 2005 [11]	*		*	*	*		*	*		******
Nöthlings et al. 2008 [25]	*	*	*	*	*		*	*		*******
Taunk et al. 2016 [26]	*	*	*		*	*	*	*		*******
Chan et al. 2005 [27]	*		*	*	*		*	*	*	*******
Shigihara et al. 2014 [28]	*	*	*		*	*	*	*		*******
Lyon et al. 1993 [29]	*			*	*		*	*		*****
Michaud et al. 2003 [30]	*	*	*		*		*	*		******
Nkondjock et al. 2005 [31]	*		*	*	*		*	*		******
Vrieling et al. 2009 [32]	*	*	*	*	*		*	*	*	********
Larsson et al. 2006 [14]	*		*	*	*		*	*		*******
Jansen et al. 2011 [33]	*		*		*		*	*		*****
Heinen et al. 2012 [34]	*	*	*		*		*	*		******
Nöthlings et al. 2005 [35]	*	*	*	*	*		*	*		*******
Stolzenberg-Solomon et al. 2007 [36]	*	*	*	*	*		*	*		*******
Larsson et al. 2006 [37]	*	*	*		*		*	*	*	*******
Anderson et al. 2002 [38]	*			*	*		*	*		*****
Inoue-Choi et al. 2011 [39]	*	*	*	*	*		*	*	*	********
Arem et al. 2013 [40]	*	*	*		*	*	*	*		*******
Olsen et al. 1989 [41]	*			*	*		*	*		*****
Silverman et al. 1995 [42]	*		*	*	*		*	*		******
Lucenteforte et al. 2012 [43]	*			*	*		*	*		*****
Heinen et al. 2009 [19]	*	*	*		*	*	*	*		*******
Tavani et al. 1997 [44]	*			*	*		*			*****
Michaud et al. 2010 [45]	*		*	*	*		*	*		******
Villeneuve et al. 2000 [46]	*		*	*	*		*	*		******
Jiao et al. 2009 [47]	*	*	*		*		*	*	*	*******
Rahman et al. 2015 [48]	*		*	*	*		*	*	*	*******
Gapstur et al. 2011 [49]	*	*	*		*		*	*		******
Michaud et al. 2001 [50]	*	*	*		*		*	*		******
Johansen et al. 2009 [51]	*	*	*		*		*	*		******

* For case-control studies, 1 indicates cases independently validated; 2 cases are representative of population; 3 community controls; 4 controls have no history of blood pressure disease; 5A study controls for age; 5B study controls for additional factor(s); 6 ascertainment of exposure by blinded interview or record; 7 same method of ascertainment used for cases and controls; and 8 non response rate the same for cases and controls. For cohort studies, 1 indicates exposed cohort truly representative; 2 non exposed cohort drawn from the same community; 3 ascertainment of exposure; 4 outcome of interest not present at start; 5A cohorts comparable on basis of age; 5B cohorts comparable on other factor(s); 6 quality of outcome assessment; 7 follow-up long enough for outcomes to occur; and 8 complete accounting for cohorts.
